# Invasive Monitoring of Cerebral Autoregulation in Aneurysmal Subarachnoid and Intracerebral Hemorrhage: A Systematic Review and Meta-Analysis

**DOI:** 10.3390/diagnostics16182918

**Published:** 2026-09-10

**Authors:** Bartosz Rodziewicz, Justyna Małgorzata Fercho, Mikołaj Kacperski, Mateusz Fidut, Emilia Wilkowska, Jacek Szypenbejl, Mariusz Siemiński

**Affiliations:** 1 Scientific Circle of Neurotraumatology, Department of Emergency Medicine, Medical University of Gdansk, 80-210 Gdansk, Poland; m.kacperski@gumed.edu.pl (M.K.); m.fidut@gumed.edu.pl (M.F.); emiliawilkowska@gumed.edu.pl (E.W.); jacek.szypenbejl@gumed.edu.pl (J.S.); 2 Department of Emergency Medicine, Medical University of Gdansk, 80-210 Gdansk, Poland; mariusz.sieminski@gumed.edu.pl; 3 Neurosurgery Department, 10th Military Research Hospital and PolyClinic SPZOZ, 85-681 Bydgoszcz, Poland; 4 Lung Transplant Unit, Cardiac Surgery Department, Medical University of Gdansk, 80-210 Gdansk, Poland

**Keywords:** cerebral autoregulation, pressure reactivity index, optimal cerebral perfusion pressure, subarachnoid hemorrhage, intracerebral hemorrhage, neurocritical care, delayed cerebral ischemia, prognosis

## Abstract

**Background:** Aneurysmal subarachnoid hemorrhage (aSAH) and spontaneous intracerebral hemorrhage (ICH) are associated with secondary brain injury. Impaired cerebral autoregulation (CA) is hypothesized to be associated with this pathophysiology, reflecting a transition toward a pressure-passive cerebrovascular state. Static clinical variables do not adequately capture this dynamic process, highlighting a potential role for continuous physiological monitoring. **Objective:** To systematically evaluate the prognostic utility of invasively monitored continuous pressure reactivity index (PRx) and deviation from optimal cerebral perfusion pressure (ΔCPPopt) in adult patients with aSAH and ICH. **Methods:** A PRISMA-compliant systematic review and meta-analysis was conducted across four major databases. Nineteen studies (13 aSAH, 6 ICH) utilizing continuous invasive monitoring were included. Primary endpoints were mortality and functional recovery; delayed cerebral ischemia (DCI) was a secondary outcome for aSAH. Data were synthesized using random-effects models. **Results:** In the ICH cohort, elevated mean PRx (Mean Difference [MD] = 0.18; *p* = 0.005) and deviations below optimal perfusion pressure (ΔCPPopt MD = −6.92 mmHg; *p* = 0.0001) were associated with unfavorable functional recovery. Furthermore, higher PRx values were significantly associated with mortality (MD = 0.21; *p* = 0.0006). For aSAH, higher PRx values correlated with poor functional outcomes (MD = 0.10; *p* = 0.04); however, this association was not robust to sensitivity analysis. Elevated PRx also correlated with DCI occurrence (MD = 0.05; *p* = 0.04), but the clinical magnitude of this difference remains uncertain. The GRADE certainty of evidence for all assessed outcomes was low, primarily due to the observational design of the included studies and the inherent risk of residual confounding. **Conclusions:** Low-certainty evidence suggests that continuous monitoring of PRx and ΔCPPopt may offer prognostic utility regarding ischemic complications and functional outcomes in spontaneous brain hemorrhage. While these findings support exploring individualized, autoregulation-guided neurocritical care, prospective multicenter validation remains necessary prior to routine clinical implementation.

## 1. Introduction

Aneurysmal subarachnoid hemorrhage (aSAH) remains a highly disabling stroke subtype associated with persistently poor functional recovery despite aneurysm securement and advances in contemporary neurocritical care [1,2]. Spontaneous intracerebral hemorrhage (ICH) follows a similarly unfavorable clinical course; notably, remote diffusion-weighted imaging lesions, identified in approximately one-quarter of patients with acute ICH, are associated with nearly a twofold increase in the odds of poor 3-month functional outcomes [3,4]. Static admission variables provide only limited insight into the ongoing progression of secondary neurologic injury, thereby leaving substantial prognostic uncertainty [5,6]. The inability of conventional baseline parameters to adequately capture this multifactorial and evolving late-deterioration phenotype highlights the need for continuous physiologic surrogates reflecting cerebrovascular integrity.

Mortality and long-term disability following aSAH and ICH are largely driven by a cascade of secondary brain injury processes evolving over the subsequent two weeks. In aSAH, delayed cerebral ischemia (DCI) and angiographic vasospasm represent the principal clinical complications. DCI affects approximately half of treated patients and remains a major determinant of poor 6-month functional outcomes [7,8]. Importantly, its pathophysiology extends beyond proximal arterial narrowing and also involves microcirculatory dysfunction and concomitant impairment of cerebrovascular autoregulation [9,10,11,12]. Similarly, secondary injury progression following ICH is mediated by hematoma expansion and perihematomal edema formation. These processes are further aggravated by elevated intracranial pressure (ICP) and impaired autoregulation, with disturbances in pressure reactivity reported to persist for up to 12 days after large-volume hemorrhage [3,4,6].

Cerebral autoregulation (CA) refers to the homeostatic capacity of cerebral resistance vessels to maintain relatively stable cerebral blood flow (CBF) across a physiologic range of cerebral perfusion pressure (CPP). Following hemorrhagic injury, impaired CA is associated with an increasingly pressure-passive cerebrovasculature, wherein CBF demonstrates greater dependence on systemic mean arterial pressure (MAP) [6,13]. The reference invasive metric for assessing cerebrovascular reactivity is the pressure reactivity index (PRx), defined as a moving correlation coefficient between intracranial pressure and MAP; the nadir of PRx across the CPP range is used to estimate the patient-specific optimal CPP (CPPopt) [13,14]. Although the prognostic utility of PRx has been extensively investigated in other forms of acute brain injury, emerging evidence suggests that its behavior in spontaneous hemorrhagic stroke may reflect a distinct and potentially more complex pathophysiological profile, thereby requiring condition-specific validation [11,15].

While cerebral autoregulation can also be assessed non-invasively, intermodality concordance remains limited, supporting why PRx is widely regarded as the invasive reference metric for assessing cerebrovascular pressure reactivity [16,17]. Despite three decades of investigation, evidence linking impaired CA to hard clinical outcomes in spontaneous hemorrhagic stroke remains fragmented. Existing data are derived predominantly from single-center observational cohorts characterized by limited sample sizes and monitoring durations that are frequently insufficient for robust CPPopt estimation [14,16,17,18]. Furthermore, a prior systematic review by Al-Kawaz identified only 5 ICH-specific studies compared with 18 aSAH reports, underscoring the relative scarcity of evidence in spontaneous ICH [6].

In addition, substantial methodological heterogeneity persists across the literature. Although the reference calculation of PRx is conventionally based on a 5-min moving correlation between averaged slow waves of MAP and ICP, studies have applied heterogeneous and non-interchangeable thresholds for defining autoregulatory failure [10,14,17]. Secondary complications—including DCI, hematoma expansion, and perihematomal edema—have likewise been inconsistently defined and temporally assessed across cohorts [3,4,8,19]. In the context of aSAH, endpoints such as clinical DCI, angiographic vasospasm, and structural cerebral infarction manifest through divergent pathophysiological mechanisms [7,8,10]. The primary data demonstrate that the included cohorts utilized heterogeneous diagnostic criteria for these ischemic events, precluding their interchangeable application when correlating autoregulatory failure with secondary brain injury [3,4,8,19]. To our knowledge, no multicenter quantitative synthesis has comprehensively evaluated the association between CA impairment and clinical outcomes across these two distinct hemorrhagic stroke populations.

Therefore, this systematic review and meta-analysis independently evaluates the prognostic utility of impaired cerebral autoregulation, specifically assessed using continuous invasive monitoring of the pressure reactivity index (PRx) and its derivative metric, optimal cerebral perfusion pressure deviation (ΔCPPopt), in adult patients with aSAH and spontaneous ICH. The primary outcomes are all-cause mortality and long-term functional outcome. Secondary outcomes include delayed cerebral ischemia (DCI) in patients with aSAH.

## 2. Methods

### 2.1. Study Design and Registration

**Figure 1:**
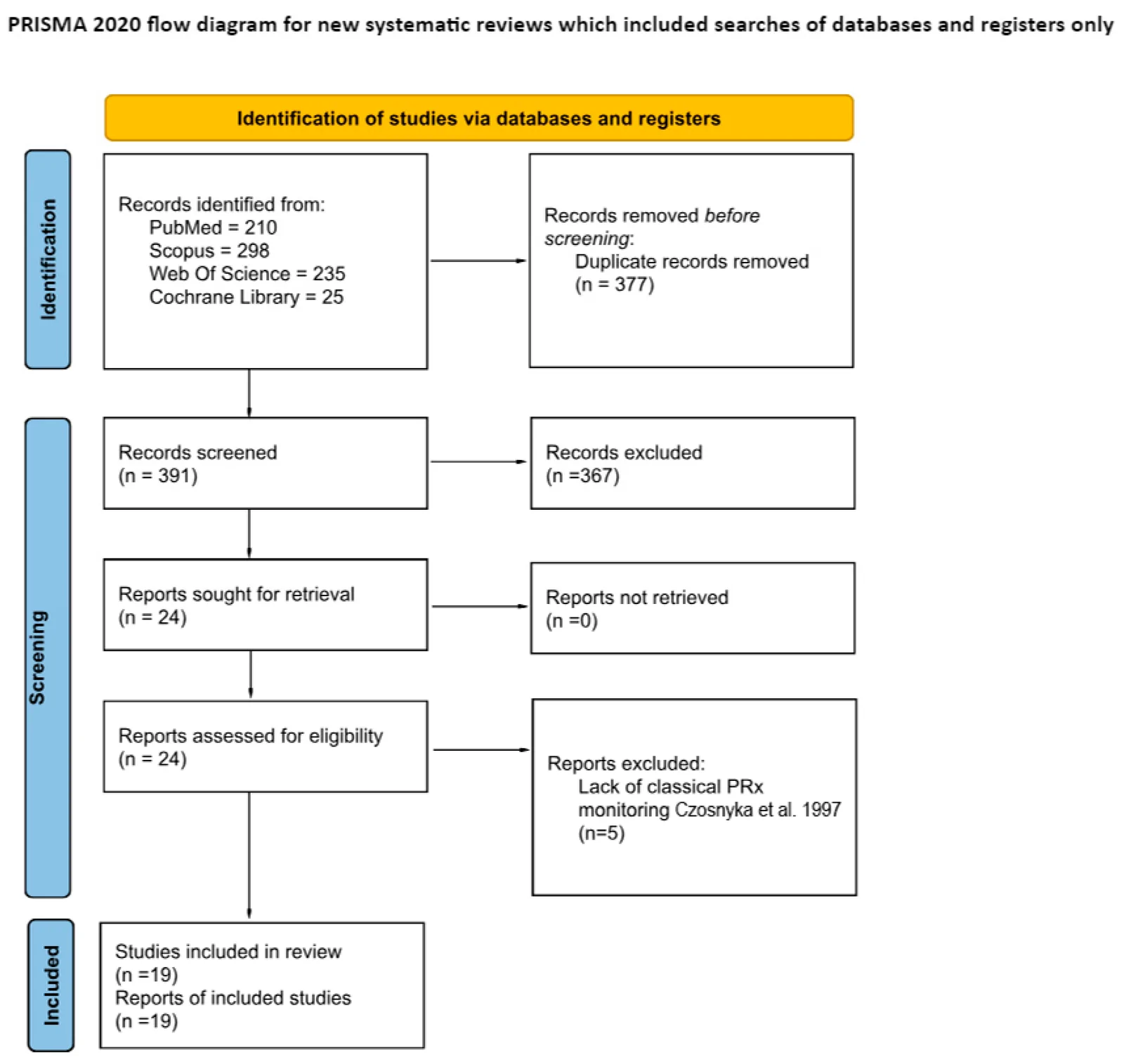
PRISMA 2020 flow diagram of study selection [21].

This systematic review and meta-analysis was conducted and reported in strict accordance with the Preferred Reporting Items for Systematic Reviews and Meta-Analyses (PRISMA) [20] guidelines (File S1). Flow diagram of inluded studies is summarized in Figure 1. The review protocol was prospectively registered in the International Prospective Register of Systematic Reviews (PROSPERO) under the registration number: CRD420261455608.

### 2.2. Search Strategy and Data Sources

A comprehensive systematic literature search was executed to identify studies evaluating the prognostic value of invasive continuous cerebral autoregulation monitoring in spontaneous hemorrhage. The search was conducted across four major electronic databases: PubMed, Web of Science (WoS), Scopus, and the Cochrane Library. The search strategy utilized a combination of free-text keywords queried across title and abstract fields. The search strings encompassed three core conceptual categories: (1) target population (“Subarachnoid Hemorrhage”, “Intracerebral Hemorrhage”, “aSAH”, “ICH”), (2) physiological metric (“Pressure Reactivity Index”, “PRx”, “optimal CPP”, “CPPopt”), and (3) clinical outcomes (“mortality”, “prognosis”, “delayed cerebral ischemia”, “outcome”). To ensure sensitivity, no temporal restrictions were applied, encompassing literature from database inception up to 10 July 2026. The search was restricted to human subjects and articles published in English. Publication types such as narrative reviews, editorials, meeting abstracts lacking full texts, and case reports (N < 10) were systematically excluded. All identified records were imported into the Rayyan reference manager, in which automated deduplication was performed [22]. The exact search strings utilized for each database are provided in the Supplementary Material (File S2).

### 2.3. Study Selection and Eligibility Criteria

Following deduplication, a two-stage screening process (title/abstract screening followed by full-text review) was performed independently by three investigators (B.R., M.F., and E.W.), yielding a Fleiss’ kappa coefficient of 0.8 for the initial title and abstract screening phase. Discrepancies regarding study eligibility were resolved through a senior experienced researcher (J.F.). Studies were deemed eligible if they met the following predefined criteria summarized in Table 1:

Crucially, studies assessing cerebral autoregulation exclusively via non-invasive modalities (e.g., Near-Infrared Spectroscopy [NIRS], Transcranial Doppler [TCD/Mx], or transient hyperemic response tests) were strictly excluded to preserve methodological homogeneity. Studies focusing on pediatric populations, traumatic brain injury (TBI), ischemic stroke, or hemorrhage secondary to tumors or vascular malformations were also excluded.

### 2.4. Data Extraction

Data extraction was performed independently by two reviewers (B.R., M.F.) to retrieve study characteristics (author, year, country, center status, sample size), patient baseline data (mean age, admission Glasgow Coma Scale (GCS)), and applied monitoring metrics (PRx, CPP, CPPopt, ΔCPPopt, ICP, ICP/PRx doses). Extracted prognostic endpoints included functional scales (GOS, GOS-E, mRS), survival status (alive vs. dead), and neurological complications (infarction, DCI). Data synthesis focused on physiological parameters stratified by clinical status: mean PRx, mean ΔCPPopt, and percentage of monitoring time (%GMT) PRx > 0.2 for favorable versus unfavorable outcomes, mean PRx for survival, and mean PRx for DCI+ versus DCI− in aSAH cohorts. To facilitate quantitative synthesis, cohort outcomes were broadly dichotomized into “favorable” versus “unfavorable” groups. Depending on the primary endpoint of the individual study, this stratification represented either long-term functional recovery (mRS 0–3 or GOS-E 5–8 vs. mRS 4–6 or GOS-E 1–4), survival status (alive vs. dead), or the absence versus presence of secondary neurological injuries (non-infarct vs. infarct, or DCI− vs. DCI+). Principal reported prognostic associations were also systematically recorded for each included study.

### 2.5. Risk of Bias Assessment

**Figure 2:**
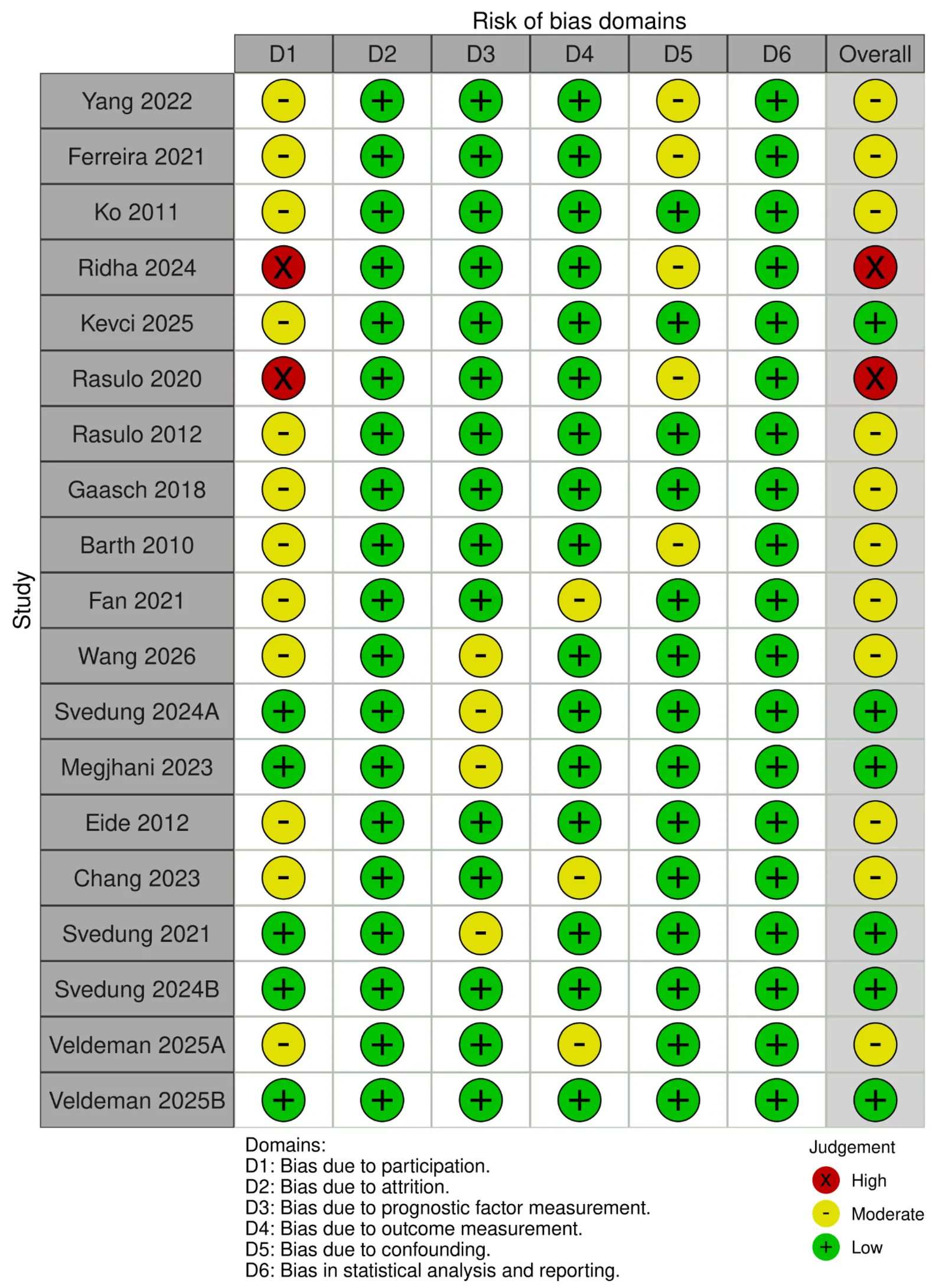
Risk of bias across the 19 included studies (QUIPS tool) [3,11,15,24,25,26,27,28,29,30,31,32,33,34,35,36,37,38,39].

The methodological risk of bias of the included prognostic studies was systematically evaluated using the Quality in Prognosis Studies (QUIPS) tool [23]. Risk of bias was appraised across six domains: study participation, study attrition, prognostic factor measurement (specifically assessing the rigor of PRx derivation), outcome measurement, study confounding, and statistical analysis and reporting. Each domain was rated as having a high, moderate, or low risk of bias. The overall risk of bias profile is summarized in Figure 2.

### 2.6. Data Synthesis and Statistical Analysis

Quantitative data synthesis was performed where at least three studies reported the same outcome using compatible metrics. To prevent double-counting of patients from overlapping institutional cohorts in the pooled effect estimates, we evaluated study origins and enrollment periods. When multiple publications derived from the same institutional database reported on the same primary or secondary endpoint, only the study with the largest sample size was included in that specific meta-analysis model.

To ensure clinical and methodological comparability for quantitative pooling, studies were required to meet the following criteria: (1) strict isolation of a single hemorrhagic pathology (aSAH or ICH); (2) uniform utilization of the classical continuous PRx derivation method (moving correlation between MAP and ICP) as defined by Czosnyka et al. [21]; and (3) reporting of compatible continuous physiological data (mean and standard deviation) stratified by harmonized, dichotomized clinical endpoints. Because variations in monitoring duration and threshold definitions persisted, pooling was strictly restricted to raw continuous variables (mean PRx or ΔCPPopt) rather than categorizations based on arbitrary, study-specific pathological thresholds.

As the extracted data for the primary variables (PRx, %GMT, and CPPopt) were continuous, pooled effect estimates were calculated as Mean Differences (MD) with corresponding 95% Confidence Intervals (CIs) using the inverse-variance weighting method. The MD was utilized because all included studies measured PRx (a correlation coefficient between −1 and 1) and ΔCPPopt (mmHg) on identical, standardized scales using the classical derivation method, ensuring direct comparability despite varying monitoring periods. Given the anticipated clinical and methodological heterogeneity—arising from variations in patient cohorts, monitoring durations, and measurement protocols—a random-effects model was employed a priori for all meta-analyses.

Statistical heterogeneity across the included studies was evaluated using Cochran’s Q statistic (with a *p*-value < 0.10 indicating statistical significance) and quantified by the I^2^ statistic. An I^2^ value greater than 50% was considered indicative of substantial heterogeneity. For analyses with ≥4 studies, 95% prediction intervals (PIs) were calculated using R version 4.5.2 (R Foundation for Statistical Computing, Vienna, Austria). To demonstrate the sources of statistical heterogeneity and assess the robustness of the pooled estimates, a leave-one-out sensitivity analysis was performed for all meta-analyses comprising more than three studies.

We a priori planned to assess potential publication bias through the visual inspection of funnel plots and Egger’s regression test. However, because fewer than 10 studies were included in each specific meta-analysis, these assessments were not performed, in accordance with the Cochrane Handbook recommendations, as such tests lack sufficient power to distinguish chance from real asymmetry in small study pools.

All statistical meta-analyses and the generation of forest plots were conducted using Review Manager (RevMan) software (version 5.4, The Cochrane Collaboration, London, UK, 2020). A two-sided *p*-value < 0.05 was considered statistically significant for overall effect estimates. A conceptual schematic diagram illustrating the underlying pathophysiological mechanisms and the systematic workflow was created using BioRender (BioRender.com). Google Gemini 3.1 Pro (Google LLC, Mountain View, CA, USA) was utilized for linguistic editing; all content was thoroughly reviewed and verified by the authors, who take full responsibility for the output.

### 2.7. Certainty of Evidence

The overall certainty of evidence for each primary outcome was evaluated using the Grading of Recommendations Assessment, Development and Evaluation (GRADE) [40] framework adapted for prognostic factor research. Evidence quality was downgraded for risk of bias, inconsistency, indirectness, imprecision, or publication bias, and could be upgraded for large effect sizes or clear dose–response gradients (worsening outcomes with progressively higher PRx values). GRADE assessment is available in Supplementary Material (File S3).

## 3. Results

### 3.1. Study Selection

The initial systematic literature search across the four electronic databases yielded a total of 768 records. Following the removal of 377 duplicates, 391 unique articles underwent title and abstract screening. Of these, 367 records were excluded for failing to fulfill the predefined eligibility criteria (primarily due to exclusive reliance on non-invasive monitoring, non-target patient populations, or preclinical study designs). Subsequently, 24 articles were retrieved for comprehensive full-text evaluation. During this phase, 5 studies were excluded due to a lack of classical PRx monitoring (as defined by Czosnyka et al. [21]). Ultimately, 19 studies met all inclusion criteria, encompassing approximately 1398 aSAH patients (after accounting for overlapping institutional cohorts) and 336 ICH patients, and were incorporated into the final systematic review.

### 3.2. Characteristics of Included Studies

The review incorporated a total of 19 studies, comprising 6 analyses of patients with intracerebral hemorrhage (ICH) [3,24,25,26,27,28] and 13 investigating aneurysmal subarachnoid hemorrhage (aSAH) cohorts [11,15,29,30,31,32,33,34,35,36,37,38,39]. The vast majority of these were single-center studies, with only two utilizing a multi-center design. Geographically, the literature was diverse, originating predominantly from European centers (including Sweden, Germany, Italy, Portugal, Austria, and Norway), as well as from the United States and China. In the reviewed studies, continuous hemodynamic monitoring was frequently expanded to assess derived parameters, including optimal cerebral perfusion pressure (CPPopt) and deviations from this target (ΔCPPopt). Several investigations also incorporated cumulative burden metrics, such as the percentage of monitoring time spent above critical thresholds (%GMT), and intracranial pressure (ICP) dose, alongside other physiological variables such as brain tissue oxygenation (PbtO_2_). Long-term functional outcomes were primarily evaluated using the classic or extended Glasgow Outcome Scale (GOS/GOS-E) and the modified Rankin Scale (mRS). These functional endpoints were often supplemented by direct survival analyses. Furthermore, within the aSAH subpopulation, particular emphasis was placed on clinical and radiological complications, specifically the incidence of delayed cerebral ischemia (DCI) and the subsequent development of secondary infarctions. Detailed data extraction for all included studies is provided in the Supplementary Material (Files S4 and S5).

### 3.3. Risk of Bias

Using the QUIPS tool, overall risk of bias among the 19 included studies was rated as low in 6/19, moderate in 11/19, and high in 2/19. The shift toward moderate risk was primarily driven by limitations in study participation—specifically, the high prevalence of single-center cohorts with small sample sizes (N < 100)—as well as challenges in confounding variable management. Additionally, minor methodological concerns resulting in moderate risk ratings were noted in prognostic factor measurement and outcome measurement across a subset of the cohorts. Conversely, all 19 investigations demonstrated a uniformly low risk of bias regarding both study attrition and statistical analysis and reporting.

### 3.4. Certainty of Evidence (GRADE)

Assessment using the GRADE framework resulted in a uniformly low level of certainty, reflecting the initial observational nature of the included cohorts. The evidence for mortality prediction, cumulative autoregulatory burden (%GMT), and delayed cerebral ischemia (DCI) was rated as LOW (⊕⊕◯◯). For mortality and %GMT, this was primarily driven by serious risk of bias-notably, 13 of the 19 included studies were judged to have a moderate or high risk, largely due to their single-center observational design and statistical inconsistency secondary to variable PRx thresholds and differing outcome timelines, whereas the evaluation of DCI was downgraded strictly due to serious imprecision arising from extremely small subgroup sample sizes (n < 10 in several contributing cohorts), which inherently compromises statistical power. For all primary and secondary endpoints, including functional outcomes in both the ICH and aSAH cohorts, the overall certainty of evidence remained LOW (⊕⊕◯◯). In accordance with the GRADE framework, despite statistically significant and directionally consistent associations across several analyses, the observational nature of the included neurocritical care cohorts carries an inherently high risk of residual confounding (e.g., severity of initial injury and variations in ICU management protocols). Therefore, strict criteria for upgrading the certainty based on the magnitude of effect were not met. For specific outcomes, such as functional outcomes in aSAH, evidence was further constrained by substantial statistical heterogeneity (I^2^ = 87%) and sensitivity analyses demonstrating that the pooled effect was statistically fragile (File S3).

### 3.5. Key Findings of Reviewed Data

In the intracerebral hemorrhage (ICH) cohorts, an association between impaired cerebral autoregulation and both mortality and unfavorable functional outcome scores was reported by Yang et al., Ferreira et al., and Rasulo et al. [24,25,28]. Several studies described PRx thresholds—most commonly exceeding 0.20 or 0.25—in relation to occurrences of brain tissue hypoxia and secondary ischemic lesions [25,26,27]. Additionally, data extracted from these cohorts indicated that a longer duration of autoregulatory impairment, when recorded alongside an elevated intracranial pressure burden, was correlated with clinical deterioration [3,28].

Within the aneurysmal subarachnoid hemorrhage (aSAH) literature, impaired autoregulation was investigated in conjunction with the incidence of delayed cerebral ischemia (DCI) and structural infarction [30,32,38,39]. In multi-center and single-center data, cumulative hypoperfusion (ΔCPPopt < −5 or −10 mmHg) was frequently documented in patients who also presented with reduced baseline autoregulatory capacity [15,32,34]. Regarding temporal patterns, autoregulatory alterations measured within the initial 72-h window [30,36] as well as impairments recorded between days 6.5 and 10 [37] were separately correlated with lower long-term functional recovery scores. Furthermore, statistical analyses in one cohort presented a U-shaped distribution, where both extreme cerebrovascular hyperreactivity and severe impairment were recorded in patients with unfavorable outcomes [11]. Finally, measurements indicating acute recovery of pressure reactivity following DCI therapies were noted alongside higher rates of 1-year functional independence [39].

### 3.6. Meta-Analysis of Clinical Outcomes

**Figure 3:**
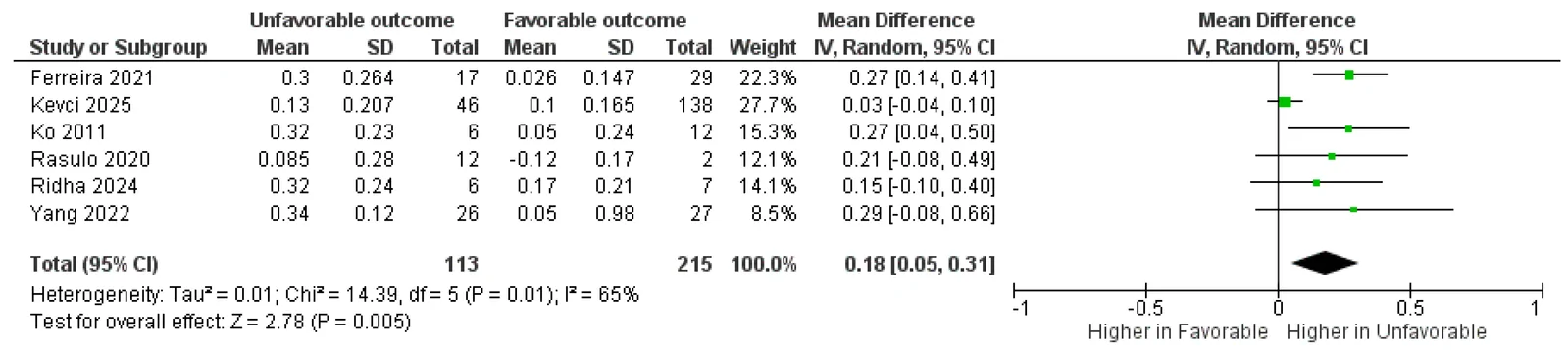
PRx and functional outcome in intracerebral hemorrhage (ICH) [3,24,25,26,27,28].

**Figure 4:**
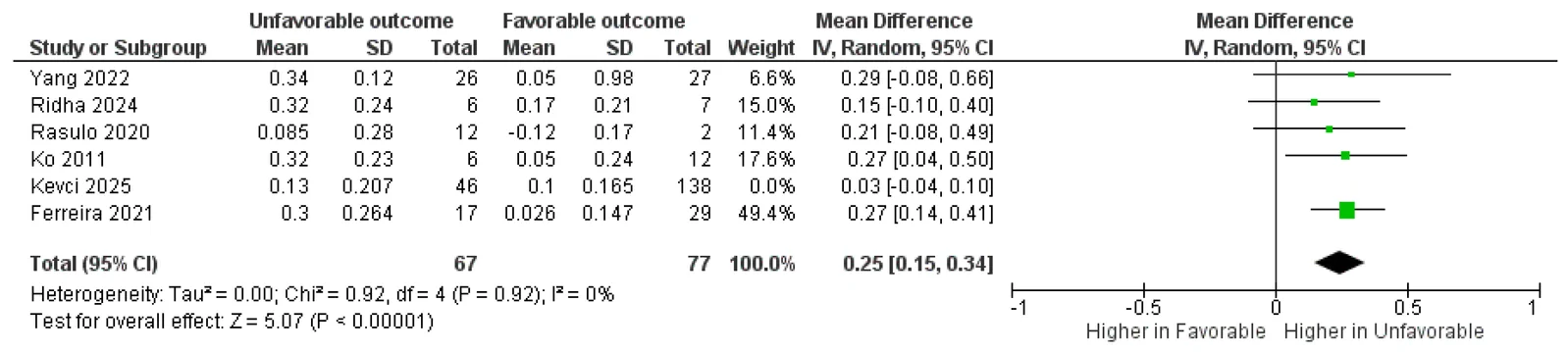
PRx and functional outcome in intracerebral hemorrhage (ICH)—leave-one-out sensitivity analysis [3,24,25,26,27,28].

**Figure 5:**
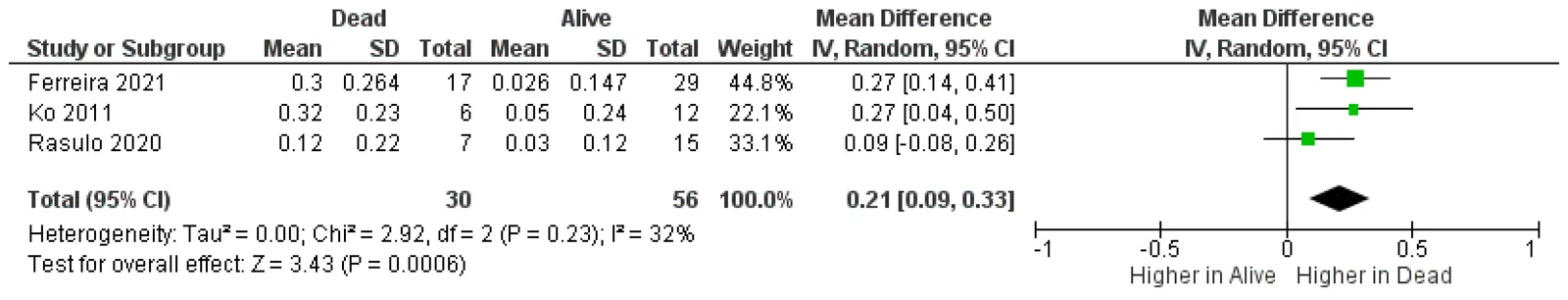
PRx and mortality in intracerebral hemorrhage (ICH) [25,26,28].

**Figure 6:**
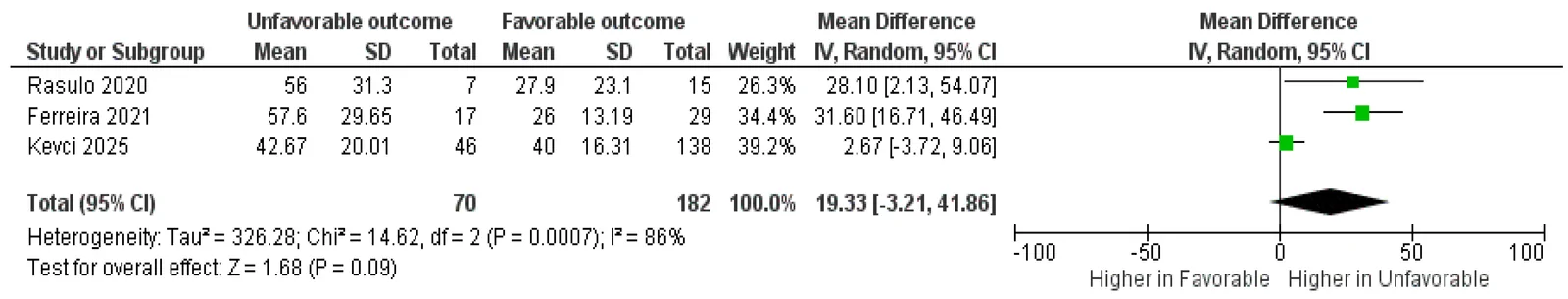
PRx %GMT and Outcome in Intracerebral Hemorrhage (ICH) [3,25,28].

**Figure 7:**
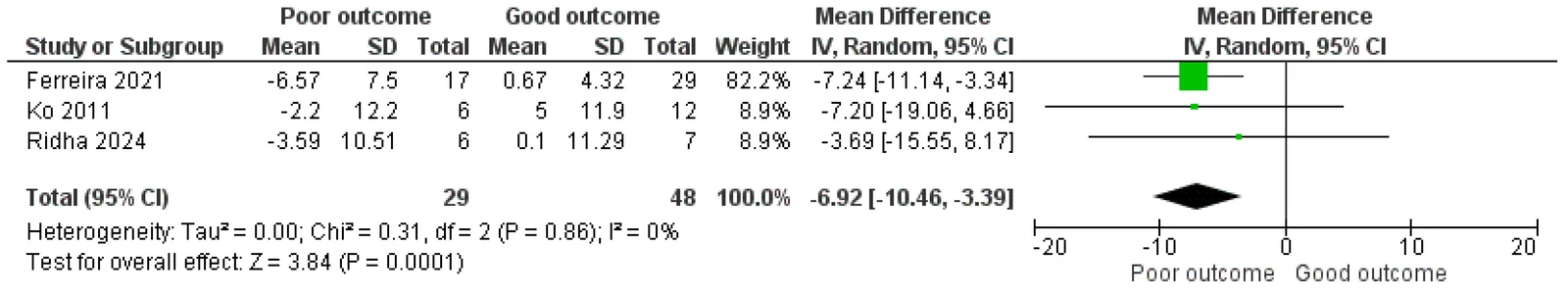
ΔCPPopt and Outcome in Intracerebral Hemorrhage (ICH) [25,26,27].

In the intracerebral hemorrhage (ICH) cohort, higher mean PRx values were observed in patients with a poor outcome compared to those with a good outcome (MD = 0.18; 95% CI: 0.05 to 0.31; 95% prediction interval [PI]: −0.15 to 0.51; *p* = 0.005; Figure 3), with moderate-to-high heterogeneity (I^2^ = 65%). Leave-one-out analysis of the ICH functional outcome model showed that exclusion of Kevci 2025 [3] increased the pooled estimate (MD = 0.25; 95% CI: 0.15 to 0.34; *p* < 0.00001) and removed statistical heterogeneity (I^2^ 65% → 0%), indicating that this single large cohort accounted for the observed inconsistency (Figure 4). Furthermore, the mortality analysis revealed a statistically significant difference, with non-survivors exhibiting significantly higher PRx values compared to survivors (MD = 0.21; 95% CI: 0.09 to 0.33; *p* = 0.0006; Figure 5), showing low-to-moderate heterogeneity (I^2^ = 32%). The evaluation of the percentage of time spent above the PRx threshold (%GMT) showed no statistically significant differences between the outcome groups (MD = 19.33; 95% CI: −3.21 to 41.86; *p* = 0.09; Figure 6), with substantial between-study heterogeneity (I^2^ = 86%). In contrast, a statistically significant difference was found for ΔCPPopt, with lower values in patients with poor outcome (MD = −6.92; 95% CI: −10.46 to −3.39; *p* = 0.0001; Figure 7), with no detectable heterogeneity (I^2^ = 0%).

**Figure 8:**
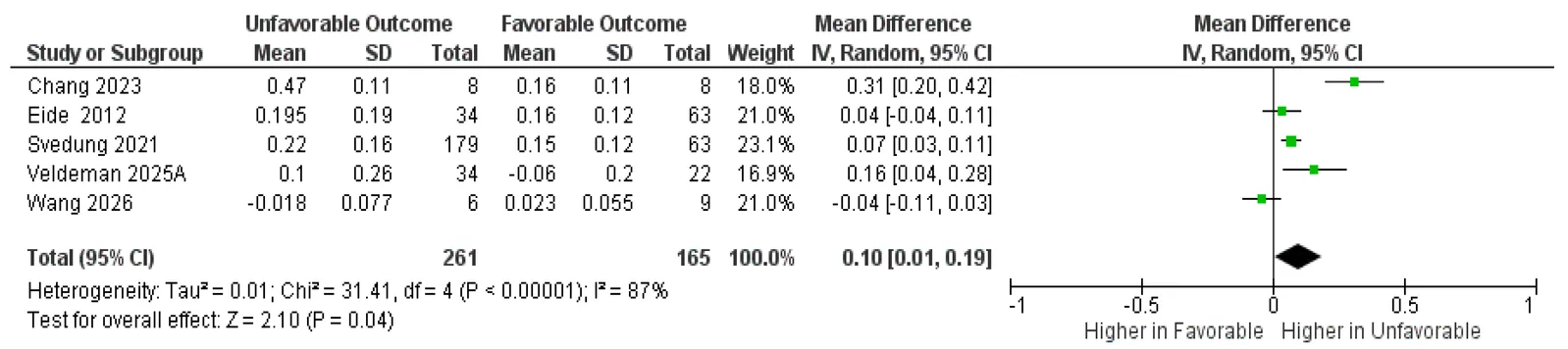
PRx and functional outcome in aneurysmal subarachnoid hemorrhage (aSAH) [33,35,36,37,38].

**Figure 9:**
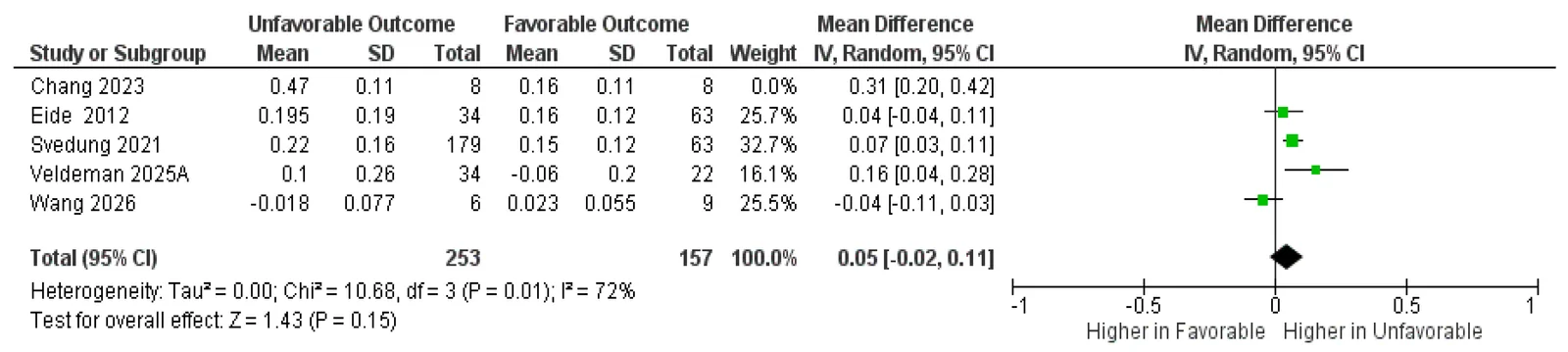
PRx and functional outcome in aneurysmal subarachnoid hemorrhage (aSAH)—leave-one-out sensitivity analysis [33,35,36,37,38].

In the aSAH cohort, higher PRx values were associated with poor outcome (MD = 0.10; 95% CI: 0.01 to 0.19; 95% PI: −0.25 to 0.45; *p* = 0.04; Figure 8), with high statistical heterogeneity (I^2^ = 87%). A leave-one-out sensitivity analysis was conducted to evaluate the robustness of the aSAH functional outcome model and explore the high statistical heterogeneity (I^2^ = 87%). Systematic exclusion of Chang et al. 2023 [36] resulted in loss of statistical significance (MD = 0.05; 95% CI: −0.02 to 0.11; *p* = 0.15) and only partially reduced heterogeneity (I^2^ = 72%; Figure 9). This indicates that the overall significant association between elevated PRx and unfavorable outcomes in the aSAH cohort is statistically fragile and primarily driven by a single highly influential study.

**Figure 10:**
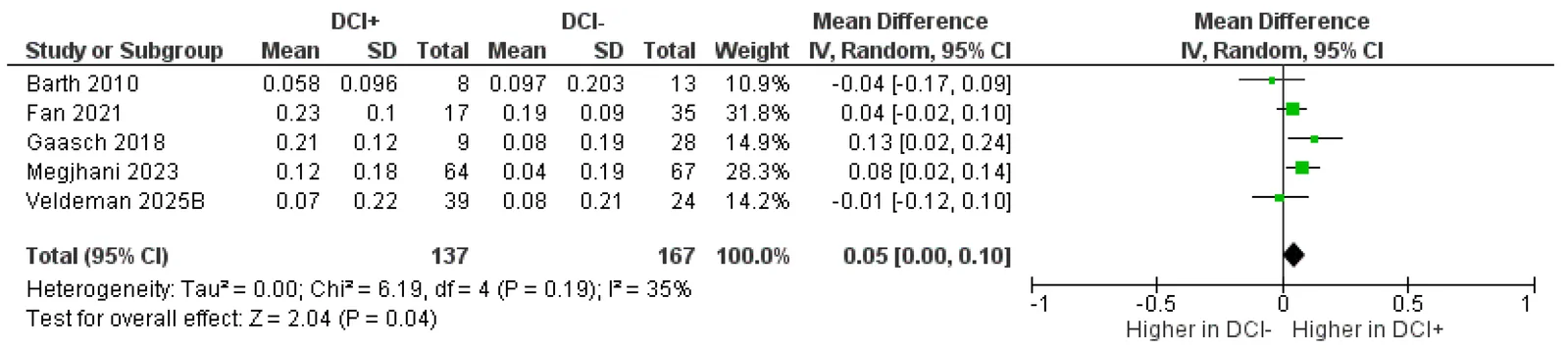
PRx and Delayed Cerebral Ischemia (DCI) in SAH [30,31,32,34,39].

Furthermore, in the assessment of secondary complications, patients who developed delayed cerebral ischemia (DCI+) exhibited higher mean PRx values compared to the cohort without this complication (DCI−) (MD = 0.05; 95% CI: 0.00 to 0.10; PI: −0.04 to 0.16; *p* = 0.04; Figure 10). Statistical heterogeneity for this specific comparison was found to be low-to-moderate (I^2^ = 35%).

**Figure 11:**
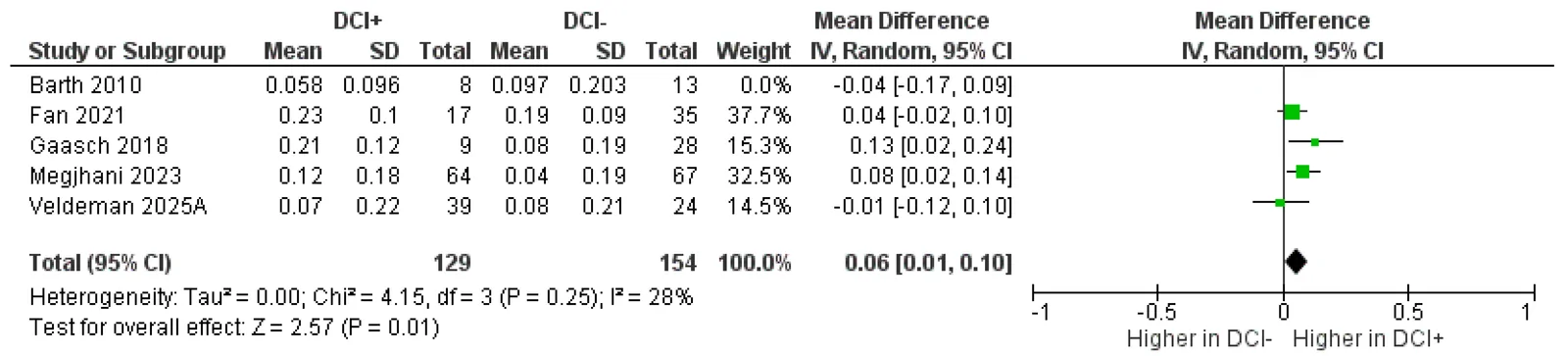
PRx and Delayed Cerebral Ischemia (DCI) in SAH— leave-one-out sensitivity analysis [30,31,32,34,38].

A leave-one-out sensitivity analysis for aSAH DCI, sequentially excluding Barth 2010 [31], increased statistical significance and reduced between-study variance (MD = 0.06; 95% CI: 0.01 to 0.10; *p* = 0.01; I^2^ = 28%) (Figure 11).

## 4. Discussion

### 4.1. Beyond Static Metrics: The Core Prognostic Signal

This synthesis suggests that continuous invasive autoregulation monitoring offers potential prognostic value extending beyond conventional static variables. Importantly, our quantitative meta-analysis indicates that impaired pressure reactivity may be associated with unfavorable clinical trajectories across both hemorrhage subtypes. In the ICH cohort, higher mean PRx values (MD = 0.18; *p* = 0.005) and larger deviations below individualized optimal perfusion pressure (ΔCPPopt MD = −6.92 mmHg; *p* = 0.0001) were associated with unfavorable functional recovery. Crucially, the corresponding analysis for mortality also demonstrated that elevated PRx was linked to non-survival (MD = 0.21; 95% CI: 0.09 to 0.33; *p* = 0.0006). These findings are consistent with individual cohort observations by Ferreira et al. [25] and Fan et al. [32]. Within the aSAH population, elevated PRx was linked to overall poor functional outcomes (MD = 0.10; 95% CI: 0.01 to 0.19; *p* = 0.04); on the other hand, this result proved statistically fragile and lost significance upon sensitivity analysis. However, the DCI analysis should be interpreted according to both statistical significance and magnitude of effect. The pooled difference in PRx between patients with and without DCI was relatively small (MD = 0.05, 95% CI: 0.00–0.10; *p* = 0.04). Although statistically significant, the lower confidence limit approaches zero, and it remains unclear whether a difference of this magnitude has meaningful discriminatory or clinical value.

These pooled findings align closely with established neurotrauma literature (e.g., Czosnyka et al. [21], Zeiler et al. [41]), suggesting that cerebrovascular dysregulation may represent a common pathophysiological mechanism across acute brain injuries. Furthermore, these results highlight the potential limitations of relying strictly on universal physiological thresholds, such as the static CPP targets of 60–70 mmHg currently advocated by AHA/ASA and Neurocritical Care Society consensus guidelines [42,43,44]. The extracted data imply that the cumulative burden of deviations from a patient-specific CPPopt may provide complementary prognostic information regarding ischemic complications and long-term disability alongside isolated pressure spikes. Consequently, transitioning from static parameters to continuous autoregulation indices may support future individualized perfusion strategies.

### 4.2. Divergent Pathways of Injury: aSAH vs. ICH

Although impaired cerebral autoregulation might predict adverse outcomes in both pathologies, the underlying secondary injury pathways appear to diverge significantly. In ICH, impaired PRx correlates with poor outcomes when coupled with high intracranial pressure (ICP dose), as suggested by several cohorts [3,24,25,45,46]. Furthermore, severely impaired pressure reactivity (e.g., PRx > 0.20 or 0.25) is associated with an increased risk of brain tissue hypoxia and remote ischemic lesions [26,27]. Conversely, in aSAH, the prognostic utility of PRx may be partly related to DCI and other secondary ischemic processes. When autoregulation fails, the brain may become exquisitely vulnerable to flow-dependent insults. Current literature [32,34] indicates that cumulative hypoperfusion (CPP < CPPopt) may serve as a robust marker associated with DCI and poor recovery, potentially acting synergistically with broader secondary ischemic mechanisms [45,47]. Furthermore, early autoregulatory derangements combined with tissue hypoxia may help differentiate reversible ischemic deficits from permanent infarction [38].

### 4.3. Timing and Tipping Points: Decoding PRx Thresholds

While historically established within the traumatic brain injury (TBI) literature, identifying pathological PRx thresholds points toward a potential clinically relevant threshold between 0.20 and 0.25 [48,49]. Recent observational data in spontaneous brain hemorrhage increasingly align with this range, suggesting that exceeding this limit may signify a transition to a pressure-passive state potentially associated with tissue hypoxia and ischemic lesion expansion [25,26,27]. However, the prognostic relationship appears to follow a U-shaped trajectory, where both severe impairment and extreme hyperreactivity are correlated with unfavorable outcomes [11,50]. Furthermore, the temporal profile of risk may be biphasic. Early derangements within 72 h [30,36] could exacerbate acute injuries, whereas late-phase impairment around days 6.5–10 [37] might be independently associated with long-term disability, potentially mirroring established secondary ischemia windows.

### 4.4. Precision Neurocritical Care: Individualized CPP Targets: A Hypothesis for Prospective Testing

**Figure 12:**
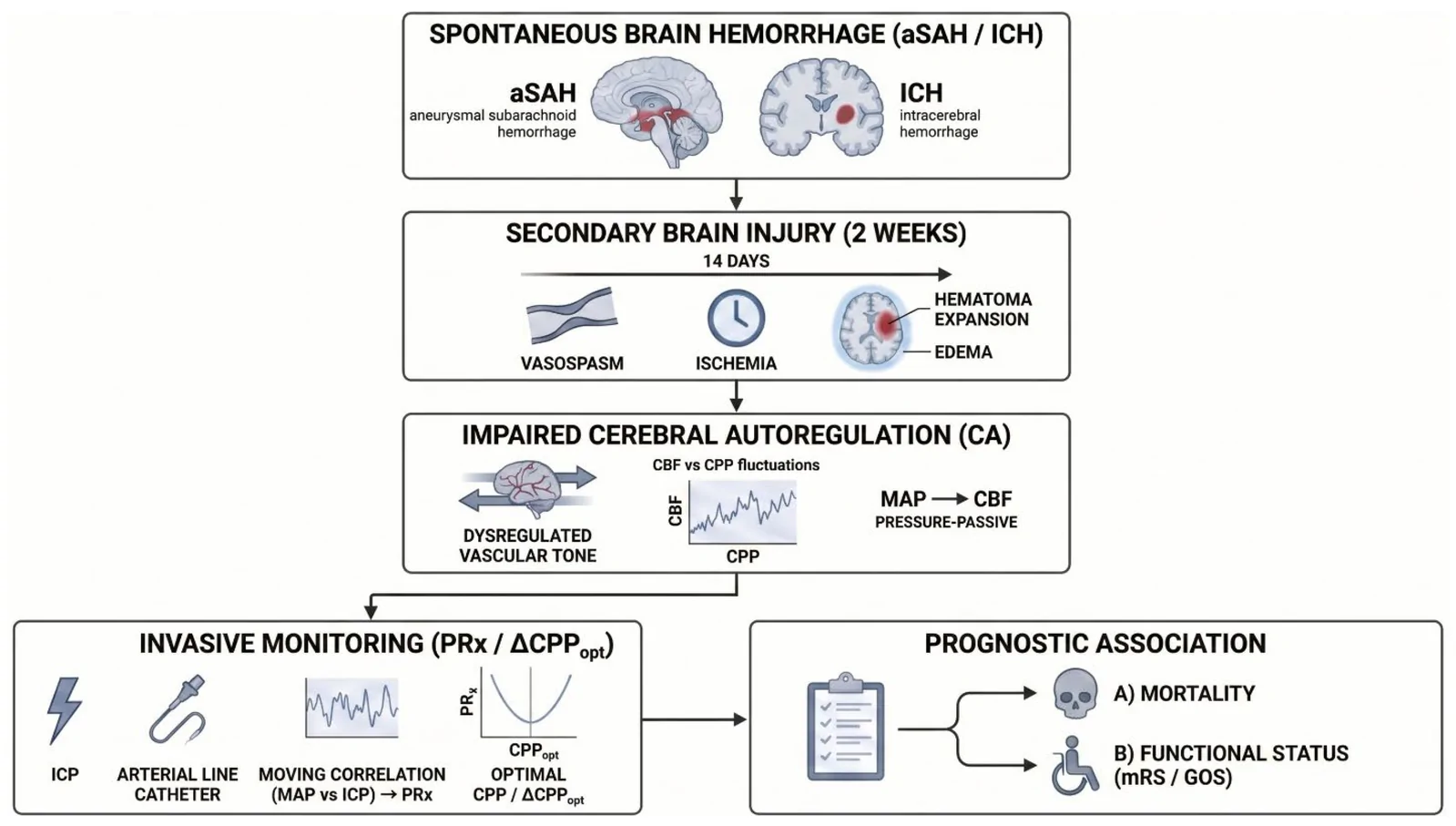
Pathophysiological workflow of secondary brain injury and invasive continuous autoregulation monitoring in aneurysmal subarachnoid (aSAH) and intracerebral hemorrhage (ICH). The 14-day secondary injury cascade may contribute to the development of a pressure-passive cerebrovascular state. Derivation of the pressure reactivity index (PRx) and optimal cerebral perfusion pressure (CPPopt/ΔCPPopt) through MAP and ICP correlations may offer prognostic utility for assessing mortality and functional recovery (mRS/GOS). Rodziewicz, B. (2026). Mapping Cerebral Autoregulation: PRx and Outcome After aSAH and ICH. Created in BioRender. https://BioRender.com/g6c9d1m (accessed on 7 September 2026).

The clinical trajectory following spontaneous brain hemorrhage involves a cascade of secondary injuries frequently accompanied by impaired cerebral autoregulation [3,4,6,8,13]. Continuous invasive monitoring—deriving the pressure reactivity index (PRx) from the moving correlation between mean arterial pressure and intracranial pressure—enables the estimation of a patient-specific optimal cerebral perfusion pressure (CPPopt) [13,14]. Automated tracking of deviations from this individualized target via a rolling 4-h window [51] offers a promising monitoring approach for assessing dynamic prognostic associations, demonstrating that relative hypoperfusion has been reported in individual cohorts to be associated with mortality, whereas hyperperfusion has been associated with severe disability [25,32,34,51]. The proposed pathophysiological workflow of this review is visually summarized in the graphic below (Figure 12).

### 4.5. Limitations

The clinical utility of these findings is constrained by several limitations inherent to the current literature, categorized into four primary domains: **Methodological and Statistical Heterogeneity**: The lack of consensus on pathological thresholds—spanning fixed cut-offs to calculated CPPopt targets—hinders protocol standardization. Furthermore, the comparability of the pooled data is limited by inherent clinical differences in exposure definitions, primarily the heterogeneous monitoring durations across the included cohorts. This is compounded by the lack of uniform diagnostic criteria for ischemic complications. Individual cohorts independently evaluated diverse endpoints—ranging from clinical DCI to angiographic vasospasm and imaging-confirmed infarction. Due to the distinct clinical trajectories of these events, analyzing them as a uniform ischemic outcome limits the precision of correlating specific PRx deviations with secondary ischemia. This methodological fragmentation is also reflected in the substantial statistical heterogeneity observed in several of our meta-analytical comparisons (e.g., I^2^ = 87% for PRx outcomes in aSAH). This variance is compounded by highly unstable monitoring durations across cohorts, compromising prognostic precision. Leave-one-out sensitivity analyses explored heterogeneity in comparisons exceeding three studies; notably, this revealed that the association between elevated PRx and poor functional outcomes in aSAH is statistically fragile, losing significance upon the exclusion of a single influential study (Chang et al., 2023 [36]). Additionally, 95% prediction intervals calculated for analyses with a sufficient number of studies (k ≥ 4) were wide and consistently crossed zero, objectively reflecting this substantial between-study variance. This instability, combined with limited study numbers that preclude meta-regression, underscores the imperative for cautious interpretation of current prognostic estimates and highlights the critical need for larger, standardized multicenter datasets. Furthermore, most retrospective studies fail to control for key physiological confounders (such as PaCO_2_ fluctuations, sedation depth, and temperature management), potentially overestimating the independent prognostic value of PRx. Additionally, there is an inability to perform publication bias analyses owing to the limited number of studies. Several pooled comparisons rely on extremely small sample sizes (e.g., n < 10 patients in specific subgroups), which compromises statistical power and limits the robustness of the effect estimates. Furthermore, the standardization of varied functional scales (mRS and GOS-E) into binary “favorable” and “unfavorable” categories introduces clinical heterogeneity, as the precise clinical significance of these cut-off points is not strictly equivalent. Due to the limited number of studies, a sensitivity analysis restricting inclusion to a single, uniformly defined functional scale was not possible.

**Selection Bias and Generalizability**: The available evidence exhibits high institutional concentration, with foundational data and methodologies originating from a few specialized academic centers. This selection bias limits the external validity and direct extrapolability of our findings to community-based or lower-resource hospitals operating under different neurocritical care standards. Additionally, restricting the eligibility criteria strictly to English-language publications introduces a potential language bias, which may have excluded relevant evidence published in other languages. **Barriers to Clinical Translation**: Unlike the cleaned datasets used in retrospective research, real-time bedside monitoring is highly vulnerable to signal artifacts from routine clinical care. This artifactual noise can generate misleading perfusion targets in a live ICU setting, highlighting the critical need for prospective, multicenter external validation before clinical implementation. **Confounding by Indication:** This synthesis relies on observational data, restricting all findings to correlational associations. Because invasive neuromonitoring is selectively indicated for patients with severe baseline neurological deficits, impaired pressure reactivity may primarily serve as a surrogate marker of the initial injury magnitude rather than an independent driver of secondary complications. Consequently, the overall GRADE certainty of evidence for all evaluated outcomes is low, indicating that future robustly designed studies are very likely to have an important impact on our confidence in these effect estimates.

## 5. Conclusions

Based on low-certainty evidence, continuous invasive monitoring of cerebral autoregulation via PRx and ΔCPPopt appears to hold potential prognostic value for assessing the risk of ischemic complications and long-term functional impairment in patients with both aneurysmal subarachnoid (aSAH) and intracerebral hemorrhage (ICH), though the association with long-term functional impairment in aSAH remains statistically fragile. Specifically, while elevated PRx showed a statistically significant correlation with DCI in aSAH, the small effect size and borderline statistical significance warrant cautious clinical interpretation. These metrics may support the design of prospective trials testing autoregulation-guided perfusion targets; whether such management improves outcome remains untested. However, the current evidence base remains largely limited to single-center observational cohorts, which are characterized by substantial methodological heterogeneity and a lack of universally accepted clinical thresholds. Consequently, despite being mechanistically plausible, the routine clinical implementation of PRx-directed therapies cannot be recommended at this stage. Future prospective, multicenter trials are necessary to validate these individualized physiological targets and to ascertain whether their application can safely and effectively improve clinical outcomes.

## Figures and Tables

**Table 1:** PECOS Criteria.

PECOS Parameter	Inclusion Criteria
Population (P)	Patients aged (≥16 years) with confirmed aneurysmal subarachnoid hemorrhage (aSAH) or primary intracerebral hemorrhage (ICH) requiring admission to an intensive care unit.
Exposure (E)	Utilization of continuous invasive multimodal monitoring to assess cerebral autoregulation, specifically reporting on the Pressure Reactivity Index (PRx) or optimal cerebral perfusion pressure (ΔCPPopt).
Comparator (C)	Comparison of clinical endpoints between patients with intact versus impaired autoregulation, or across varying strata of PRx/ΔCPPopt deviations.
Outcomes (O)	Primary endpoints: all-cause mortality or functional outcome assessed via the modified Rankin Scale (mRS), Glasgow Outcome Scale (GOS), or Extended GOS (GOS-E). Secondary endpoints: Neurological complications including angiographic vasospasm, delayed cerebral ischemia (DCI), hematoma expansion, or perihematomal edema.
Study Design (S)	Prospective and retrospective observational cohorts, case–control studies, and secondary analyses of randomized controlled trials (RCTs).

## Data Availability

The datasets generated and analyzed during the current review are available within the article and its Supplementary Material files. Further raw data extractions are available from the corresponding authors upon reasonable request.

## Supplementary Materials

The following supporting information can be downloaded at: https://www.mdpi.com/article/10.3390/diagnostics16182918/s1, File S1: PRISMA 2020 Checklist; File S2: Search Strategies Across Databases; File S3: GRADE Evidence Profile; File S4: Data Extraction Table—Intracerebral Hemorrhage (ICH); File S5: Data Extraction Table—Aneurysmal Subarachnoid Hemorrhage (aSAH).

## Abbreviations

The following abbreviations are used in this manuscript:

aSAH	Aneurysmal subarachnoid hemorrhage
CA	Cerebral autoregulation
CBF	Cerebral blood flow
CPP	Cerebral perfusion pressure
CPPopt	Optimal cerebral perfusion pressure
ΔCPPopt	Deviation from optimal cerebral perfusion pressure
DCI	Delayed cerebral ischemia
GOS	Glasgow Outcome Scale
GOS-E	Extended Glasgow Outcome Scale
GRADE	Grading of Recommendations Assessment, Development and Evaluation
ICH	Intracerebral hemorrhage
ICP	Intracranial pressure
MAP	Mean arterial pressure
mRS	Modified Rankin Scale
PbtO_2_	Brain tissue oxygenation
PRx	Pressure reactivity index
QUIPS	Quality in Prognosis Studies
TBI	Traumatic brain injury

## References

[1] Ezra M., Garry P., Rowland M.J., Mitsis G.D., Pattinson K.T. (2021). Phase dynamics of cerebral blood flow in subarachnoid haemorrhage in response to sodium nitrite infusion. Nitric Oxide.

[2] Chang J.J., Dowlati E., Felbaum D.R., Mai J.C. (2022). Relationship of pressure reactivity index and delayed cerebral ischemia in aneurysmal subarachnoid hemorrhage. Turk. Neurosurg..

[3] Kevci R., Hånell A., Howells T., Fahlström A., Lewén A., Enblad P., Svedung Wettervik T. (2025). Temporal dynamics of ICP, CPP, PRx, and CPPopt in relation to outcome in spontaneous intracerebral hemorrhage. J. Neurosurg..

[4] Posener S., Hmeydia G., Benzakoun J., Oppenheim C., Baron J.C., Turc G. (2023). Remote diffusion-weighted imaging lesions and intracerebral hemorrhage: A systematic review and meta-analysis. Stroke.

[5] Sen R.D., McGrath M.C., Shenoy V.S., Meyer R.M., Park C., Fong C.T., Lele A.V., Kim L.J., Levitt M.R., Wang L.L. (2025). A dynamic machine learning model to predict angiographic vasospasm after aneurysmal subarachnoid hemorrhage. Neurosurgery.

[6] Al-Kawaz M., Cho S.M., Gottesman R.F., Suarez J.I., Rivera-Lara L. (2022). Impact of cerebral autoregulation monitoring in cerebrovascular disease: A systematic review. Neurocrit. Care.

[7] Hernández-Durán S., Mielke D., Rohde V., Malinova V. (2019). Does nimodipine interruption due to high catecholamine doses lead to a greater incidence of delayed cerebral ischemia in the setting of aneurysmal subarachnoid hemorrhage?. World Neurosurg..

[8] Rostami E., Engquist H., Howells T., Johnson U., Ronne-Engström E., Nilsson P., Hillered L., Lewén A., Enblad P. (2018). Early low cerebral blood flow and high cerebral lactate: Prediction of delayed cerebral ischemia in subarachnoid hemorrhage. J. Neurosurg..

[9] Conzen C., Albanna W., Weiss M., Kürten D., Vilser W., Kotliar K., Zäske C., Clusmann H., Schubert G.A. (2018). Vasoconstriction and impairment of neurovascular coupling after subarachnoid hemorrhage: A descriptive analysis of retinal changes. Transl. Stroke Res..

[10] Carlson A.P., Jones T., Zhu Y., Desai M., Alsarah A., Shuttleworth C.W. (2025). Oxygen-based autoregulation indices associated with clinical outcomes and spreading depolarization in aneurysmal subarachnoid hemorrhage. Neurocrit. Care.

[11] Svedung Wettervik T., Howells T., Hånell A., Lewén A., Enblad P. (2024). The optimal pressure reactivity index range is disease-specific: A comparison between aneurysmal subarachnoid hemorrhage and traumatic brain injury. J. Clin. Monit. Comput..

[12] Malinova V., Kranawetter B., Tuzi S., Moerer O., Rohde V., Mielke D. (2024). Interaction of optimal cerebral perfusion pressure with early brain injury and its impact on ischemic complications and outcome following aneurysmal subarachnoid hemorrhage. Neurocrit. Care.

[13] Kranawetter B., Tuzi S., Moerer O., Mielke D., Rohde V., Malinova V. (2024). Optimal cerebral perfusion pressure during induced hypertension and its impact on delayed cerebral infarction and functional outcome after subarachnoid hemorrhage. Sci. Rep..

[14] Johnson U., Engquist H., Howells T., Nilsson P., Ronne-Engström E., Lewén A., Rostami E., Enblad P. (2016). Bedside xenon-CT shows lower CBF in SAH patients with impaired CBF pressure autoregulation as defined by pressure reactivity index (PRx). Neurocrit. Care.

[15] Svedung Wettervik T.M., Hånell A., Howells T., Ronne-Engström E., Lewén A., Enblad P. (2024). Individualized autoregulation-derived cerebral perfusion targets in aneurysmal subarachnoid hemorrhage: A new therapeutic avenue?. J. Intensive Care Med..

[16] Rynkowski C.B., de Oliveira Manoel A.L., Dos Reis M.M., Puppo C., Worm P.V., Zambonin D., Bianchin M.M. (2019). Early transcranial Doppler evaluation of cerebral autoregulation independently predicts functional outcome after aneurysmal subarachnoid hemorrhage. Neurocrit. Care.

[17] Ku Y., Megjhani M., Alalqum T., Kwon S.B., Nametz D., Weinerman B., Velazquez A., Ghoshal S., Agarwal S., Roh D.J. (2025). The relationship between pressure reactivity and cerebral oximetry indexes in patients with aneurysmal subarachnoid hemorrhage: A single-center pilot study. Neurocrit. Care.

[18] Burzyńska M., Uryga A., Kasprowicz M., Czosnyka M., Dragan B., Kübler A. (2020). The relationship between the time of cerebral desaturation episodes and outcome in aneurysmal subarachnoid haemorrhage: A preliminary study. J. Clin. Monit. Comput..

[19] Van der Harst J.J., Elting J.W.J., Bokkers R.P.H., Veeger N.J.G.M., van Donkelaar C.E., van den Bergh W.M., Metzemaekers J.D.M., Groen R.J.M., Mazuri A., Luijckx G.R. (2023). The diagnostic value of near-infrared spectroscopy to predict delayed cerebral ischemia and unfavorable outcome after subarachnoid hemorrhage. World Neurosurg..

[20] Page M.J., McKenzie J.E., Bossuyt P.M., Boutron I., Hoffmann T.C., Mulrow C.D., Shamseer L., Tetzlaff J.M., Akl E.A., Brennan S.E. (2021). The PRISMA 2020 statement: An updated guideline for reporting systematic reviews. BMJ.

[21] Czosnyka M., Smielewski P., Kirkpatrick P., Laing R.J., Menon D., Pickard J.D. (1997). Continuous assessment of the cerebral vasomotor reactivity in head injury. Neurosurgery.

[22] Ouzzani M., Hammady H., Fedorowicz Z., Elmagarmid A. (2016). Rayyan—A web and mobile app for systematic reviews. Syst. Rev..

[23] Hayden J.A., van der Windt D.A., Cartwright J.L., Côté P., Bombardier C. (2013). Assessing bias in studies of prognostic factors. Ann. Intern. Med..

[24] Yang Y., Pan Y., Chen C., Zhao P., Hang C. (2022). Clinical significance of multiparameter intracranial pressure monitoring in the prognosis prediction of hypertensive intracerebral hemorrhage. J. Clin. Med..

[25] Ferreira A.V., Maia I., Dias C., Depreitere B., Meyfroidt G., Güiza F. (2021). Monitoring of cerebrovascular reactivity in intracerebral hemorrhage and its relation with survival. Intracranial Pressure and Neuromonitoring XVII.

[26] Ko S.B., Choi H.A., Parikh G., Helbok R., Schmidt J.M., Lee K., Badjatia N., Claassen J., Connolly E.S., Mayer S.A. (2011). Multimodality monitoring for cerebral perfusion pressure optimization in comatose patients with intracerebral hemorrhage. Stroke.

[27] Ridha M., Megjhani M., Nametz D., Kwon S.B., Velazquez A., Ghoshal S., Agarwal S., Claassen J., Roh D.J., Connolly E.S. (2024). Suboptimal cerebral perfusion is associated with ischemia after intracerebral hemorrhage. Neurocrit. Care.

[28] Rasulo F., Piva S., Park S., Oddo M., Megjhani M., Cardim D., Matteotti I., Gandolfi L., Robba C., Taccone F.S. (2020). The association between peri-hemorrhagic metabolites and cerebral hemodynamics in comatose patients with spontaneous intracerebral hemorrhage: An international multicenter pilot study analysis. Front. Neurol..

[29] Rasulo F.A., Girardini A., Lavinio A., De Peri E., Stefini R., Cenzato M., Nodari I., Latronico N. (2012). Are optimal cerebral perfusion pressure and cerebrovascular autoregulation related to long-term outcome in patients with aneurysmal subarachnoid hemorrhage?. J. Neurosurg. Anesthesiol..

[30] Gaasch M., Schiefecker A.J., Kofler M., Beer R., Rass V., Pfausler B., Thomé C., Schmutzhard E., Helbok R. (2018). Cerebral autoregulation in the prediction of delayed cerebral ischemia and clinical outcome in poor-grade aneurysmal subarachnoid hemorrhage patients. Crit. Care Med..

[31] Barth M., Woitzik J., Weiss C., Muench E., Diepers M., Schmiedek P., Kasuya H., Vajkoczy P. (2010). Correlation of clinical outcome with pressure-, oxygen-, and flow-related indices of cerebrovascular reactivity in patients following aneurysmal SAH. Neurocrit. Care.

[32] Fan B.B., Sun X.C., Huang Z.J., Yang X.M., Guo Z.D., He Z.H. (2021). Hypoperfusion assessed by pressure reactivity index is associated with delayed cerebral ischemia after subarachnoid hemorrhage: An observational study. Chin. Neurosurg. J..

[33] Wang T., Xiao Y., Liao Q., Wang F., Jiang Y., Cui S., Chen P., Huo Y., Shu J., Fan W. (2026). Impact of intracranial pressure and related parameters on prognosis in patients undergoing surgery for ruptured intracranial aneurysms. Neurosurg. Rev..

[34] Megjhani M., Weiss M., Ford J., Terilli K., Kastenholz N., Nametz D., Kwon S.B., Velazquez A., Agarwal S., Roh D.J. (2023). Optimal cerebral perfusion pressure and brain tissue oxygen in aneurysmal subarachnoid hemorrhage. Stroke.

[35] Eide P.K., Sorteberg A., Bentsen G., Marthinsen P.B., Stubhaug A., Sorteberg W. (2012). Pressure-derived versus pressure wave amplitude-derived indices of cerebrovascular pressure reactivity in relation to early clinical state and 12-month outcome following aneurysmal subarachnoid hemorrhage. J. Neurosurg..

[36] Chang J.J., Kepplinger D., Metter E.J., Felbaum D.R., Mai J.C., Armonda R.A., Aulisi E.F. (2023). Pressure reactivity index for early neuroprognostication in poor-grade subarachnoid hemorrhage. J. Neurol. Sci..

[37] Svedung Wettervik T., Howells T., Lewén A., Ronne-Engström E., Enblad P. (2021). Temporal dynamics of ICP, CPP, PRx, and CPPopt in high-grade aneurysmal subarachnoid hemorrhage and the relation to clinical outcome. Neurocrit. Care.

[38] Veldeman M., Bögli S.Y., Olakorede I., Kastenholz N., Weiss M., Conzen-Dilger C., Seyfried K., Beqiri E., Weyland C., Clusmann H. (2025). Monitoring treatment of delayed cerebral ischaemia in unconscious patients after aneurysmal subarachnoid haemorrhage: A prospective multimodal neuromonitoring study. Stroke Vasc. Neurol..

[39] Veldeman M., Bögli S.Y., Olakorede I., Kastenholz N., Weiss M., Conzen-Dilger C., Seyfried K., Beqiri E., Weyland C., Clusmann H. (2025). The metabolic and autoregulatory profile of reversible delayed cerebral ischemia in unconscious patients after aneurysmal subarachnoid hemorrhage: A prospective multimodal neuromonitoring cohort study. Crit. Care.

[40] Guyatt G.H., Oxman A.D., Vist G.E., Kunz R., Falck-Ytter Y., Alonso-Coello P., Schünemann H.J. (2008). GRADE: An emerging consensus on rating quality of evidence and strength of recommendations. BMJ.

[41] Zeiler F.A., Ercole A., Cabeleira M., Carbonara M., Stocchetti N., Menon D.K., Smielewski P., Czosnyka M. (2019). CENTER-TBI High Resolution (HR ICU) Sub-Study Participants and Investigators. Comparison of performance of different optimal cerebral perfusion pressure parameters for outcome prediction in adult traumatic brain injury: A collaborative European NeuroTrauma Effectiveness Research in Traumatic Brain Injury (CENTER-TBI) study. J. Neurotrauma.

[42] Hoh B.L., Ko N.U., Amin-Hanjani S., Chou S.H.-Y., Cruz-Flores S., Dangayach N.S., Derdeyn C.P., Du R., Hänggi D., Hetts S.W. (2023). 2023 Guideline for the management of patients with aneurysmal subarachnoid hemorrhage: A guideline from the American Heart Association/American Stroke Association. Stroke.

[43] Greenberg S.M., Ziai W.C., Cordonnier C., Dowlatshahi D., Francis B., Goldstein J.N., Hemphill J.C., Johnson R., Keigher K.M., Mack W.J. (2022). 2022 Guideline for the management of patients with spontaneous intracerebral hemorrhage: A guideline from the American Heart Association/American Stroke Association. Stroke.

[44] Diringer M.N., Bleck T.P., Claude Hemphill J., Menon D., Shutter L., Vespa P., Bruder N., Connolly E.S., Citerio G., Gress D. (2011). Critical care management of patients following aneurysmal subarachnoid hemorrhage: Recommendations from the Neurocritical Care Society’s Multidisciplinary Consensus Conference. Neurocrit. Care.

[45] Otite F., Mink S., Tan C.O., Puri A., Zamani A.A., Mehregan A., Chou S., Orzell S., Purkayastha S., Du R. (2014). Impaired cerebral autoregulation is associated with vasospasm and delayed cerebral ischemia in subarachnoid hemorrhage. Stroke.

[46] Zheng H., Chen C., Zhang J., Hu Z. (2016). Mechanism and therapy of brain edema after intracerebral hemorrhage. Cerebrovasc. Dis..

[47] Budohoski K.P., Czosnyka M., Smielewski P., Kasprowicz M., Helmy A., Bulters D., Pickard J.D., Kirkpatrick P.J. (2012). Impairment of cerebral autoregulation predicts delayed cerebral ischemia after subarachnoid hemorrhage: A prospective observational study. Stroke.

[48] Hiler M., Czosnyka M., Hutchinson P., Balestreri M., Smielewski P., Matta B., Pickard J.D. (2006). Predictive value of initial computerized tomography scan, intracranial pressure, and state of autoregulation in patients with traumatic brain injury. J. Neurosurg..

[49] Sorrentino E., Diedler J., Kasprowicz M., Budohoski K.P., Haubrich C., Smielewski P., Outtrim J.G., Manktelow A., Hutchinson P.J., Pickard J.D. (2012). Critical thresholds for cerebrovascular reactivity after traumatic brain injury. Neurocrit. Care.

[50] Lidington D., Wan H., Bolz S.S. (2021). Cerebral autoregulation in subarachnoid hemorrhage. Front. Neurol..

[51] Aries M.J., Czosnyka M., Budohoski K.P., Steiner L.A., Lavinio A., Kolias A.G., Hutchinson P.J., Brady K.M., Menon D.K., Pickard J.D. (2012). Continuous determination of optimal cerebral perfusion pressure in traumatic brain injury. Crit. Care Med..

