# Supplementary material for: Invasive Monitoring of Cerebral Autoregulation in Aneurysmal Subarachnoid and Intracerebral Hemorrhage: A Systematic Review and Meta-Analysis
Source: Diagnostics (Basel). 2026 Sep 10;16(18):2918. doi: 10.3390/diagnostics16182918 (PMC13605057; doi:10.3390/diagnostics16182918)
Supplement: Supplementary file 1 [file diagnostics-16-02918-s001.zip › FILE S2.pdf]

File S2: Search Strategies Across Databases

pubmed: ("Subarachnoid Hemorrhage" OR "Intracerebral Hemorrhage" OR "aSAH" OR "ICH") AND ("Cerebral Autoregulation" OR "Pressure Reactivity Index" OR "PRx" OR "Mx" OR "NIRS" OR "Optimal CPP") AND ("Prognosis" OR "Mortality" OR "Delayed Cerebral Ischemia" OR "Hematoma Expansion" OR "Outcome")

Scopus: TITLE-ABS-KEY (("Subarachnoid Hemorrhage" OR "Intracerebral Hemorrhage" OR "aSAH" OR "ICH") AND ("Cerebral Autoregulation" OR "Pressure Reactivity Index" OR "PRx" OR "Mx" OR "NIRS" OR "Optimal CPP") AND ("Prognosis" OR "Mortality" OR "Delayed Cerebral Ischemia" OR "Hematoma Expansion" OR "Outcome"))

WOS: ("Subarachnoid Hemorrhage" OR "Intracerebral Hemorrhage" OR "aSAH" OR "ICH") AND ("Cerebral Autoregulation" OR "Pressure Reactivity Index" OR "PRx" OR "Mx" OR "NIRS" OR "Optimal CPP") AND ("Prognosis" OR "Mortality" OR "Delayed Cerebral Ischemia" OR "Hematoma Expansion" OR "Outcome")

cochrane: ("Subarachnoid Hemorrhage" OR "Intracerebral Hemorrhage" OR "aSAH" OR "ICH") AND ("Cerebral Autoregulation" OR "Pressure Reactivity Index" OR "PRx" OR "Mx" OR "NIRS" OR "Optimal CPP") AND ("Prognosis" OR "Mortality" OR "Delayed Cerebral Ischemia" OR "Hematoma Expansion" OR "Outcome")

SCOPUS : 298
COCHRANE : 25
WOS : 235
PUBMED : 210
