# Supplementary material for: Invasive Monitoring of Cerebral Autoregulation in Aneurysmal Subarachnoid and Intracerebral Hemorrhage: A Systematic Review and Meta-Analysis
Source: Diagnostics (Basel). 2026 Sep 10;16(18):2918. doi: 10.3390/diagnostics16182918 (PMC13605057; doi:10.3390/diagnostics16182918)
Supplement: Supplementary file 1 [file diagnostics-16-02918-s001.zip › FILE S3.pdf]

File S3: GRADE Evidence Profile

Outcome (Endpoint)	No. of Studies (N total)	Risk of Bias	Inconsistency	Indirectness	Imprecision	Other Considerations	Certainty of Evidence (GRADE)
Intracerebral Hemorrhage (ICH)							
PRx & Functional Outcome (Fig 3)	6 (Observational); N = 328	Not serious [a]	Serious [b]	Not serious	Not serious	None [c]	⊕⊕○○ LOW
PRx & Mortality (Fig 4)	3 (Observational); N = 86	Serious [d]	Not serious	Not serious	Serious [e]	None	⊕⊕○○ LOW
PRx %GMT & Functional Outcome (Fig 5)	3 (Observational); N = 252	Serious [d]	Serious [f]	Not serious	Serious [g]	None	⊕⊕○○ LOW
CPPopt & Functional Outcome (Fig 6)	3 (Observational); N = 77	Serious [d]	Not serious	Not serious	Not serious	None [h]	⊕⊕○○ LOW
Aneurysmal Subarachnoid Hemorrhage (aSAH)							
PRx & Functional Outcome (Fig 7)	5 (Observational); N = 426	Not serious	Serious [i]	Not serious	Not serious	None [j]	⊕⊕○○ LOW

PRx & Delayed Cerebral Ischemia (Fig 8)	5 (Observational); N = 304	Not serious	Not serious	Not serious	Serious [k]	None	⊕⊕○○ LOW

Explanations:

a Not downgraded because high-risk studies contribute only 11% of the total weight. Excluding them would not alter the pooled estimate, which is driven primarily by moderate-risk cohorts. However, it is noted that across the overall synthesis, 13 of the 19 included studies were judged to have a moderate or high risk, largely due to their single-center observational design.

b Substantial heterogeneity ($I^2 = 65\%$) stems from varying PRx thresholds and outcome assessment timelines. However, the direction of effect—higher PRx in unfavorable outcomes—remains consistent across all cohorts.

c No upgrade applied. Although there is a statistically significant association, the observational nature of neurocritical care cohorts carries an inherent high risk of residual confounding (e.g., severity of initial injury and variations in ICU management).

d The limited number of studies (n = 3) precludes formal sensitivity analysis. One cohort carries a high risk of bias, while the remaining two are moderate, consistent with the broader finding that most included cohorts are single-center.

e The pooled effect is not statistically significant (p = 0.08) due to a small sample size (N = 86). However, the direction of effect is directionally consistent with the primary functional outcome analysis.

f High heterogeneity ($I^2 = 89\%$) reflects variable operationalization of %GMT and differing thresholds. Despite this, the direction of the association remains consistent across all three cohorts.

g The pooled effect did not reach statistical significance (p = 0.09) and crosses the null. Still, the point estimate aligns directionally with the primary PRx-outcome analysis.

h No upgrade applied. Despite a clinically meaningful deviation and zero statistical heterogeneity, the very small sample size (N = 77) across only three observational cohorts makes it impossible to confidently exclude unmeasured confounders.

i Substantial heterogeneity ($I^2 = 87\%$) is driven by differences in PRx calculations, monitoring duration, and outcome thresholds. Nonetheless, the direction of effect remains consistent across all five cohorts.

j No upgrade applied. While the association is directionally consistent, the statistical significance is modest ($p = 0.04$). In the context of observational design, this magnitude does not surpass the threshold required by the GRADE framework to rule out residual confounding and upgrade certainty.

k The statistical significance is borderline ($p = 0.04$) because the lower confidence limit abuts the null. Consequently, a minimum clinically important difference between DCI+ and DCI− patients cannot be confidently excluded.

Methodological notes

All included studies were observational by design and were therefore assigned a starting certainty rating of LOW per the GRADE working group framework adapted for prognostic factor research. Downgrading was applied for serious risk of bias, statistical inconsistency, indirectness of the population, exposure, or outcome criteria, imprecision (wide confidence intervals, non-significance, or failure to meet optimal information size), or evidence of publication bias. Assessment of publication bias by funnel plot asymmetry or Egger's regression test was not performed for any of the six pooled outcomes because fewer than ten studies contributed to each analysis, in accordance with the Cochrane Handbook recommendations.

Abbreviations

CI = confidence interval; **CPPopt** = optimal cerebral perfusion pressure; **DCI** = delayed cerebral ischemia; **GOS / GOS-E** = Glasgow Outcome Scale / Extended; **%GMT** = percentage of monitoring time above PRx threshold; **GRADE** = Grading of Recommendations, Assessment, Development and Evaluation; **ICH** = intracerebral hemorrhage; **ICP** = intracranial pressure; **MD** = mean difference; **mRS** = modified Rankin Scale; **PRx** = pressure reactivity index; **QUIPS** = Quality In Prognosis Studies tool; **aSAH** = aneurysmal subarachnoid hemorrhage; ⊕ = high-certainty component; ◯ = low-certainty component.
