# Supplementary material for: Invasive Monitoring of Cerebral Autoregulation in Aneurysmal Subarachnoid and Intracerebral Hemorrhage: A Systematic Review and Meta-Analysis
Source: Diagnostics (Basel). 2026 Sep 10;16(18):2918. doi: 10.3390/diagnostics16182918 (PMC13605057; doi:10.3390/diagnostics16182918)
Supplement: Supplementary file 1 [file diagnostics-16-02918-s001.zip › FILE S4.pdf]

File S4: Data Extraction Table – Intracerebral Hemorrhage (ICH)

Author and Year	Country(multi or single center) and cohort size	Mean Age and Admission GCS	Applied Index:	Prognostic Evaluation Tool	Mean Prx – good outcome	Mean Prx – bad outcome	Mean PRx at Death	Mean PRx in Survivors	DELTA CPPOPT- good outcome	DELTA CPPOPT- bad outcome	%GMT PRX> 0.2 – good outcome	%GMT PRX >0.2 -bad outcome	Key findings
Kevci 2025 [3]	Sweden (Single-center) 184	56.33±11; -	PRx	GCS	0.10 ± 0.165	0.13 ± 0.207	-	-	-	-	40.0 % ±16.3%	42.67±20.0%	PRx > 0.4 is not an independent predictor alone (OR = 1.00, p=0.8) , but severe autoregulatory failure (PRx > 0.5) narrows perfusion safe zones and worsens outcomes when ICP > 15 mmHg.
Yang 2022 [23]	China (Single-center) 53	64.1±12.7; [4−9]	PRx, CPP	GOS	0.05 +- 0.98	0.34 +- 0.12	-	-	-	-	-	-	Impaired PRx and ICP dose (DICP20) independently predict poor functional outcome (GOS I–III). DICP20 **:** OR = 1.346 (p=0.019); PRx (+0.01)**:** OR = 1.052 (p=0.026).
Ferreira 2021 [24]	Portugal (Single-center) 46	62.6 ± 11.8; -	PRx, ICP, CPP, CPPopt, ICP dose, PRx dose	alive/dead	0.026 ± 0.147	0.30 ± 0.264	0.30 ± 0.264	0,026 ± 0.147	0,67 ± 4.32 mm Hg	−6,57 ± 7.50 mm Hg	26.0% ±17.8 %	57.6 %±40.0%	Mean PRx > 0.25 drastically increases 28-day mortality (33-fold risk increase). PRx > 0.25: OR = 33.0 (p<0.001); PRx dose (+1%): OR = 1.06 (p=0.003).

File S4: Data Extraction Table – Intracerebral Hemorrhage (ICH)

Ko 2011 [25]	USA (Single-center) 18	54.1 ± 14.7 ; 6,00±2.96	PRX, CCP	alive/dead	0.05 ± 0.24	0.32 ± 0.23	0.32 ± 0.23	0.05 ± 0.24	5.0 ± 11.9 mm Hg	-2.2 ± 12.2 mm Hg	-	-	Impaired autoregulation (PRx > 0.2) triples the risk of brain tissue hypoxia. PRx > 0.2: OR = 3.05 (p=0.003). Survivors show significantly lower, stable PRx.
Ridha 2024 [26]	USA (Single-center) 13	58.9 ± 14.3; GCS <8	PRx, CPPopt, ΔCPPopt	GCS, mRS,	0.17 ± 0.21	0.32 ± 0.24	-	-	+0.10 ± 11.29 mm Hg	−3.59 ± 10.51 mm Hg	-	-	Patients with ischemic lesions (DWI) have higher mean PRx (0.32 vs 0.17; p=0.201). A critical threshold of PRx > 0.25defines the LLA, below which DWI risk increases 5.77-fold (p=0.002).
Rasulo 2020 [27]	Italy, USA, Switzerland (Multi-center) 22	67.88±13.61; 4.0 ± 2.0	PRx,CPPopt, CPP	GOS	- 0.12± 0.17	0.085 ± 0.28	0.12 ± 0.22	0.03 ± 0.12	-	-	27.9% ± 23.1%	56.0 % ± 31.3 %	Non-survivors exhibit significantly worse mean PRx (0.12 vs 0.03; p=0.003) and spend twice as much time with autoregulatory failure than survivors (56.0% vs 27.9%; p=0.03).

Abbreviations: PRx: Pressure Reactivity Index; CPP: Cerebral Perfusion Pressure; GOS: Glasgow Outcome Scale; DICP20: ICP dose over 20 mmHg; OR: Odds Ratio; ICP: Intracranial Pressure; CPPopt: Optimal CPP; ΔCPPopt: Deviation from Optimal CPP; mRS: modified Rankin Scale; GCS: Glasgow Coma Scale; GCS M: GCS Motor score; DWI: Diffusion-Weighted Imaging; LLA: Lower Limit of Autoregulation; %GMT: Percentage of Monitoring Time.
