# Supplementary material for: Invasive Monitoring of Cerebral Autoregulation in Aneurysmal Subarachnoid and Intracerebral Hemorrhage: A Systematic Review and Meta-Analysis
Source: Diagnostics (Basel). 2026 Sep 10;16(18):2918. doi: 10.3390/diagnostics16182918 (PMC13605057; doi:10.3390/diagnostics16182918)
Supplement: Supplementary file 1 [file diagnostics-16-02918-s001.zip › FILE S5.pdf]

File S5: Data Extraction Table – Aneurysmal Subarachnoid Hemorrhage (aSAH)

Author and Year	Country(multi or single center) and cohort size	Mean Age and Admission GCS	Applied Index:	Prognostic Evaluation Tool	Mean Prx – good outcome	Mean Prx – bad outcome	Mean prx dci+	mean prx dci-	Key findings
Rasulo 2012 [28]	Italy (Single-center) 29	56.1±14.6; 4.00 ± 5.93	PRx; CPPopt	GOS	-	-	-	-	Impaired autoregulation duration (PRx > 0.2) independently predicts poor 6-month functional outcome (OR = 1.14, p=0.010). CPP deviation duration is not significant.
Gaasch 2018 [29]	Austria (Single-center) 43	56.00± 15.56; -	PRx	mRS	-	-	0,21 ± 0.12	0,08 ± 0.19	Early impaired autoregulation (first 72h) independently predicts DCI (OR = 6.3 per 0.1 PRx increase, p=0.042) and poor 3-month outcome (OR = 14.4 per 0.1 PRx increase, p=0.002).
Barth 2010 [30]	Germany (Single-center) 21	51.48± 8.42; -	PRx	infarct/noninfarct	-	-	0.058 ± 0.096	0.097 ± 0.203.	Continuous autoregulation monitoring is feasible. Lower PRx links to better 6-month outcome (r=-0.52, p<0.05), but indices do not acute predict DCI.
Fan 2021 [31]	China (Single-center) 52	53.17± 9.73; 2.67±2.22	PRx; CPPopt	mRS	-	-	0.23 ± 0.10	0.19 ± 0.09	Severe deviations below CPPopt (ΔCPPopt < −10 mmHg: OR = 1.114, p=0.006) and dynamic hypoperfusion (PRx > 0.3 + CPP < CPPopt: OR = 1.091, p=0.026) independently increase DCI and poor 3-month risk.
Wang 2026 [32]	China (Single-center) 15	56.80± 9.70; -	CPPopt; ΔCPP; PRx	GOS	0.023 ± 0.055	-0.018 ± 0.077	-	-	Postoperative ICP > 15 mmHg (HR = 2.14, p=0.003) and cumulative hypoperfusion burden (CPP < CPPopt: HR = 1.45per 10% time increase, p=0.012) independently drive poor 6-month prognosis.
Svedung 2024A [15]	Sweden (Single-center) 432	58.00± 12.00; -	PRx; CPPopt; ΔCPPopt	GOS-E	-	-	-	-	Hypoperfusion (ΔCPPopt < -5 mmHg) worsens 1-year outcome only when autoregulation is broken (PRx > 0.25, p<0.05). Intact PRx (≤0.25) tolerates wider CPP variations.
Megjhani 2023 [33]	USA, Germany (multi-center) 131	54.83± 12.96; -	PRx; CPPopt;	mRS	-	-	0.12 ± 0.18	0.04 ± 0.19	Perfusion below optimal (ΔCPPopt < -5 mmHg: OR = 1.39, p=0.04) and tissue hypoxia (PbtO2 < 20 mmHg: OR = 1.34, p=0.04) are independent predictors of DCI.

File S5: Data Extraction Table – Aneurysmal Subarachnoid Hemorrhage (aSAH)

			ΔCPPopt						
Eide 2012 [34]	Norway (Single-center) 94	55.66± 12.41; -	PRx ; IAAC	mRS	0.16 ± 0.12	0.195 ± 0.19	-	-	PRx does not predict long-term clinical states. Neither mean PRx nor its burden correlates with 12-month GOS (p=0.091) or mortality (p=0.072), proving inferior to amplitude-derived indices (IAAC).
Chang 2023 [35]	USA (Single-center) 16	50.50± 12.54; GCS < 8	PRx	mRS	0.16 ± 0.11	0.47 ± 0.11	-	-	Early impaired autoregulation on Day 3 independently predicts poor 12-month prognosis in poor-grade aSAH (OR = 1.25per 0.1 PRx increase, p=0.019).
Svedung 2021 [36]	Sweden (Single-center) 242	58.00± 11,00; 5.33±0,74	PRx,	GOS-E	0.15± 0.12	0.22 ± 0.16	-	-	Early PRx (Days 1–3) has no predictive value. However, late impaired autoregulation (Days 6.5–10) is a massive driver of poor 6-month GOS-E (PRx: OR = 0.02 for favorable outcome, p=0.003).
Veldeman 2025A [37]	Germany (Single-center) 56	54.6 ± 11.5; -	PRx	mRS	−0.06 ± 0.20	+0.10 ± 0.26	-	-	Elevated PRx (OR = 1.58 per 0.1 increase, p=0.009) and deeper tissue hypoxia (PbtO2 decrease: OR = 1.13, p=0.021) preceding hypoperfusion differentiate permanent infarction from reversible DCI.
Svedung , 2024B [11]	Sweden (Single-center) 487	59.00± 11.85; -	PRx	GOS-E	-	-	-	-	The PRx-outcome relationship is U-shaped. Both extreme hyperreactivity (PRx 0.5 to 1.0: OR = 2.45, p<0.001) and severe impairment (PRx -1.0 to -0.5: OR = 1.76, p=0.014) predict poor 1-year prognosis.
Veldeman 2025B [38]	Germany (Single-center) 268	54.7 ± 11.8; -	PRx	DCI-related infarction	-	-	0.07 ± 0.22	0.08 ± 0.21	Post-treatment recovery of autoregulation directly dictates survival. An absolute PRx decrease ≥ 0.1 after DCI therapy is a powerful independent predictor of favorable 1-year mRS (OR = 3.12, p=0.022).

Abbreviations: PRx: Pressure Reactivity Index; DCI: Delayed Cerebral Ischemia; GCS: Glasgow Coma Scale; GOS: Glasgow Outcome Scale; GOS-E: Extended Glasgow Outcome Scale; mRS: modified Rankin Scale; CPP: Cerebral Perfusion Pressure; CPPopt: Optimal Cerebral Perfusion Pressure; ΔCPP / ΔCPPopt: Deviation from Optimal Cerebral Perfusion Pressure; PbtO2: Brain Tissue Oxygen Tension; IAAC: Intracranial Arterial Amplitude Correlation; OR: Odds Ratio; HR: Hazard Ratio; aSAH: Aneurysmal Subarachnoid Hemorrhage; ICP: Intracranial Pressure.
